# Nanonets Collect Cancer Secretome from Pericellular Space

**DOI:** 10.1371/journal.pone.0154126

**Published:** 2016-04-21

**Authors:** Rong Zhou, Yi Kuang, Jie Zhou, Xuewen Du, Jie Li, Junfeng Shi, Richard Haburcak, Bing Xu

**Affiliations:** Department of Chemistry, Brandeis University, Waltham, Massachusetts, United States of America; Stanford University, UNITED STATES

## Abstract

Identifying novel cancer biomarkers is important for early cancer detection as it can reduce mortality rates. The cancer secretome, the collection of all macromolecules secreted by a tumor cell, alters its composition compared to normal tissue, and this change plays an important role in the observation of cancer progression. The collection and accurate analysis of cancer secretomes could lead to the discovery of novel biomarkers, thus improving outcomes of cancer treatment. We unexpectedly discovered that enzyme-instructed self-assembly (EISA) of a D-peptide hydrogelator results in nanonets/hydrogel around cancer cells that overexpress ectophosphatases. Here we show that these nanonets are able to rapidly collect proteins in the pericellular space (i.e., near the surface) of cancer cells. Because the secretory substances are at their highest concentration near the cell surface, the use of pericellular nanonets to collect the cancer secretome maximizes the yield and quality of samples, reduces pre-analytical variations, and allows the dynamic profiling of secretome samples. Thus, this new approach has great potential in identifying the heterotypic signaling in tumor microenvironments thereby improving the understanding of tumor microenvironments and accelerating the discovery of potential biomarkers in cancer biology. Data are available via ProteomeXchange with identifier PXD003924.

## Introduction

**Fig 1:**
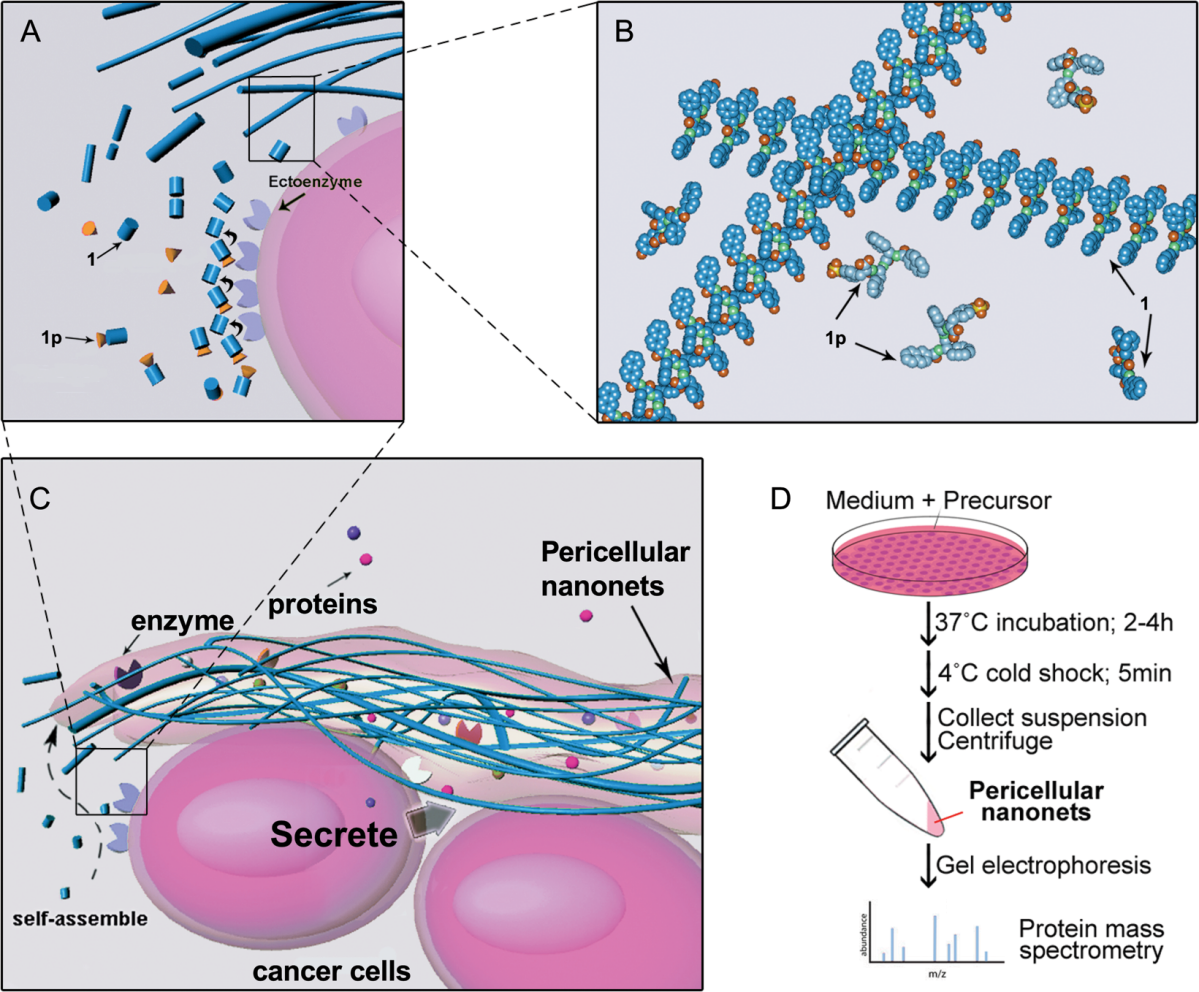
The formation of pericellular nanonets for collecting cancer secretome. (A) The dephosphorylation of the precursor turning into hydrogelator near the cell surface (i.e., pericellular space) by ectophosphatases. (B) The self-assembly of hydrogelators forming networks of nanofibrils. (C) Illustration of the sequestration of the cancer secretome by nanonets/hydrogels formed in the pericellular space of cancer cells. (D) Flow chart of the use of the pericellular nanonets/hydrogel to collect proteins in the cancer secretome.

As cancer is one of the most costly and fatal diseases for public health (e.g., NIH estimates that the overall costs of cancer in 2008 was $201.5 billion[1]), the early detection of cancer, usually resulting in less extensive treatment and better overall outcomes, is a optimal solution to significantly minimize the cost and save lives. Indeed, considerable efforts have focused on the discovery of cancer biomarkers. While the secretome is defined as the collection of all macromolecules secreted by a cell, the cancer secretome differs in composition from the secretome of normal tissues due to specific genetic mutations in cancer cells. In principle, the cancer secretome is a treasure trove of cancer biomarkers.[2] However, the definitive identification of cancer biomarkers remains a major challenge because the proteins in cancer secretome, the key soluble constituents of tumor microenvironments, [3,4] are *dynamic and complex*. Unfortunately, conventional methods (e.g., conditioned media (CM)) of collecting cancer secretome have their limits and cannot accommodate the dynamics and complexity of cancer secretome. During our research on enzyme-catalyzed formation of supramolecular nanofibrils,[5–12] we unexpectedly discovered that nanonets/hydrogel of a small D-peptide selectively form in the pericellular space of certain cancer cells due to their overexpression of ectophosphatases[12,13]. Because D-peptides resist digestion by proteases, the D-peptide based nanonets/hydrogel is stable and can trap secreted molecules (Fig 1A–1C). Thus, pericellular formation of D-peptide based nanonets/hydrogel offers a new opportunity in the sampling process by taking advantage of biological differences between cancer and normal cells.

Our study shows that within 2–4 h, the pericellular nanonets/hydrogel not only collects more of the total secreted proteins from HeLa cells than conditioned media does (e.g., media cultured with cancer cells for 24 hours), but also reduces pre-analytical variations and allows the temporal profiling of cancer secretome samples. As a powerful technique for rapid and selective collection of cancer secretome, the use of nanonets of D-peptide, thus, serves as a much-needed general sampling method in the pericellular space, where secretory substances are at their highest concentrations. Within this approach using pericellular nanonets, our investigation of the temporal profiles of cancer secretome also provides perceptive insights into the dynamic nature of cancer secretome, which may be a useful tool when used in conjunction with other quantification methods (e.g., SILAC), and eventually unveiling clinically worthwhile cancer biomarkers for the detection and treatment of cancer. In addition, the approach established in this work could help develop new methods to collect secretory signaling substances (e.g., exosomes[14] and miRNAs[15]) *in vitro* and *in vivo*, ultimately bringing new understanding to cancer biology and clinical care.

## Experimental Procedures

### Building blocks of nanonets/hydrogel

Based on our previous research on the formation of the pericellular nanofibers/hydrogel on cancer cells,[12] we used a pair of precursor/hydrogelator for the enzymatic formation of pericellular nanonets/hydrogel via self-assembly. The precursor of the hydrogelator is a naphthalene (Nap) capped tripeptide consisting of D-amino acid residues and is phosphorylated at the tyrosine residue, Nap-D-Phe-D-Phe-D-Tyr(PO_3_H_2_) (**1**_**p**_). Upon dephosphorylation, the precursor turns into Nap-D-Phe-D-Phe-D-Tyr (**1**), which self-assembles in aqueous phase to form a hydrogel.[16] The overexpression of ectophosphatases of cancer cells enables the self-assembly of **1** around the cells to form networks of nanofibrils (i.e., nanonets). In our previous work,[12] we have demonstrated that pericellular nanonets form on the surface of HeLa cells within 2h of incubation with **1**_**p**_ (400 μg/mL). We followed our previous procedure by dissolving precursor **1**_**p**_ in ddH_2_O with adjustment of pH to 7.4 by addition of 1N NaOH to produce the stock solution.

### Collection of nanonets/hydrogels or medium in complete medium

3×10^5^ of HeLa cells in 2 mL of complete MEM medium were seeded into a 35 mm Petri dish. After 24 h incubation, the medium was removed, and the cells were washed with 2 mL of fresh complete MEM medium once. For collection of nanonets/hydrogels, 1 mL of complete MEM medium containing **1**_**p**_ at 400 μg/mL (diluted from a 20X stock solution of **1**_**p**_ in PBS buffer, prepared immediately before use) was added to replace the culture medium. After 2 h incubation at 37˚C, the dish was placed in a 4˚C room for 5 min. The dish was tilted and agitated to collect the detached nanonets/hydrogels in medium. Using a wide mouth transfer pipette, we collected the medium into a 1.5 mL eppendorf tube and centrifuged at 7500 rpm for 1 minute. The suspension medium was carefully removed using a 200 μL pipette to afford the nanonets/hydrogels (Fig 1D, also two clips of video (S1 Video and S2 Video) recording the collection of nanonets and medium can be found in the supporting files) for gel electrophoresis. For collection of medium, to the cells was added 1 mL of complete MEM medium without **1**_**p**_ and the cells were incubated at 37˚C for 2 or 24 h. 100 μL of the medium was collected after incubation. For each of the experiments, the sample was obtained from the same batch of cells. Both the nanonets/hydrogels and the medium were immediately frozen at -80˚C after the collection. The additional control experiment 2h_N no cells and dynamic profiling experiments follow the same procedure for collecting nanonets/hydrogel. The details of these experiments are described in the S1 File.

### SDS-PAGE

18 μL of each sample was mixed with 12 μL of 2X Laemmli loading buffer. The solutions were mixed and incubated at 95°C for 5 min. 15μL of the solution was used for SDS-PAGE. Precast 4–20% gel in Tris-HCl (10 well, 30 μl) was used. The gel was run at a constant voltage of 200 V.

### Mass spectrometry analysis and database search

For protein mass analysis, the gel was stained by Coomassie. Each lane was cut into three sections with molecular weight ranges at: 250–80 (250–75); 80–40 (80–37); 40–10 kDa. The gel bands were excised with as little excess empty gel as possible, and were placed into a micro centrifuge tube with ddH_2_O. The samples were stored in eppendorf tubes and sent to Taplin Mass Spectrometry Facility (TMSF of Harvard Medical School) for analysis. The pictures of the gel prior to excision of gel bands were submitted (photocopy and electronically) along with the sample. The sample prepared for protein mass spectrometry is 10 μg per lane. Mass-spectrometry and protein identification were performed by TMSF.

All proteins were identified by SEQUEST (the database search algorithm, http://thompson.mbt.washington.edu/sequest) from the mass spec results and predicted fragmentation pattern of the peptide. The SEQUEST output information was provided by TMSF, including peptide sequences, XCorr (cross correlation), ∆Cn (delta correlation), number of unique and total peptides, and total spectrum counts. The SEQUEST result files are converted into valid PRIDE XML for submission to the publicly available PRIDE database by PRIDE converter[17]. The mass spectrometry proteomics data have been deposited to the ProteomeXchange Consortium via the PRIDE [18] partner repository with the dataset identifier PXD003924 and 10.6019/PXD003924.

## Results

### Time of the collection and cell compatibility of the nanonets

To select the optimal time of incubation for collecting the nanonets, HeLa cells were incubated with **1**_**p**_ for 3 to 9 h and the amount of tubulins (a marker for autolysis of cells)[19] in the nanonets was compared to the same volume (20 μL) of CM collected after 24 h of incubation. The nanonets collected after 3 or 6 h of incubation contain less tubulin than that collected after 24h CM (Fig A in S1 Fig), which suggests that the nanonets collected within 6 h of incubation contain less proteins resulting from autolysis. In another experiment, we test the viability of cells after nanonet collection (4 h incubation with **1**_**p**_) and find that cold shock and medium removal hardly induce any change in the viability of the cells (Fig B in S1 Fig). These results confirm that cold shock to collect nanonets within 6 h of incubation with the cells is suitable for collecting the secretome.

### Higher quantity and quality of the secretome collected by the nanonets

**Fig 2:**
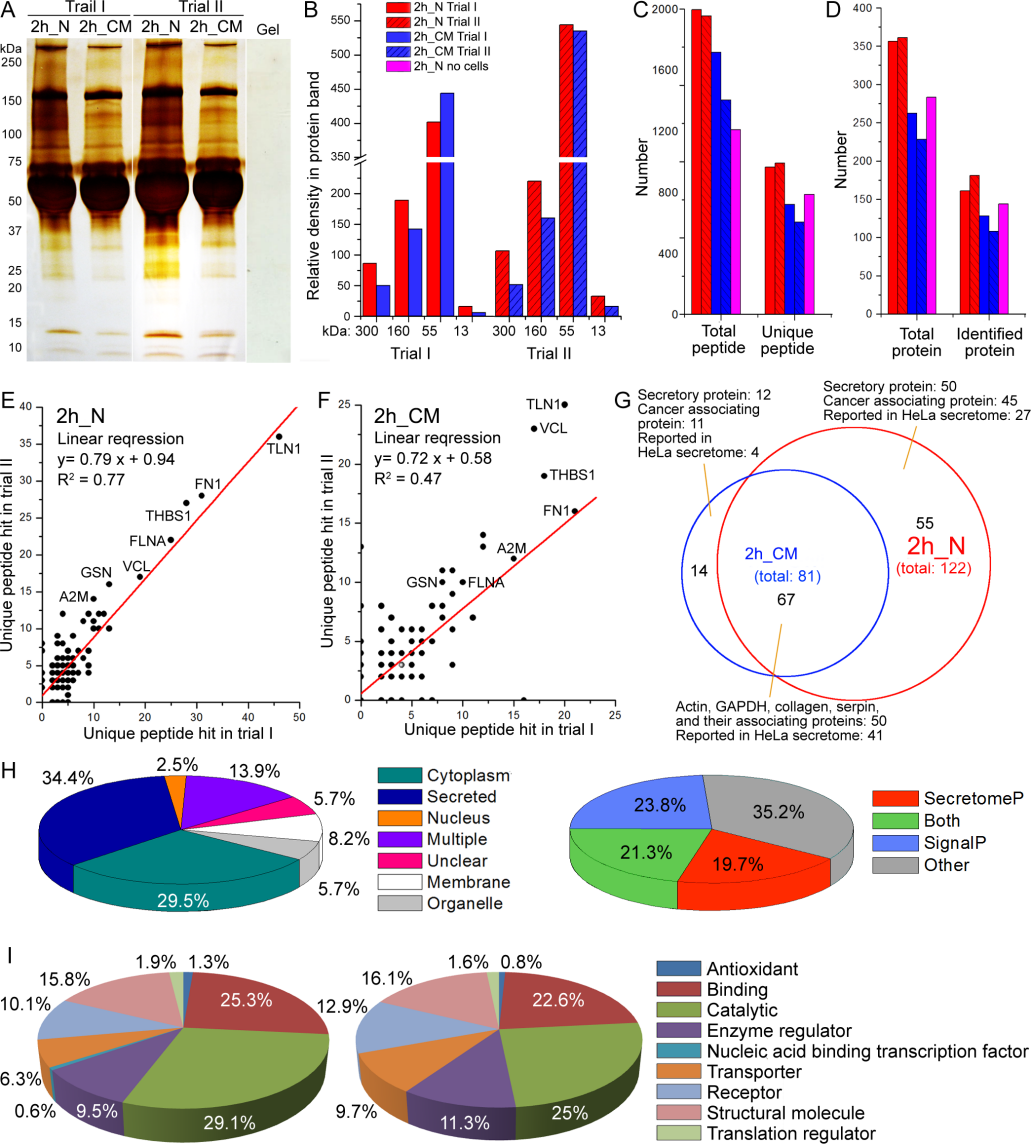
The nanonets collect more secretory proteins with lower pre-analytical variations. (A) Silver stained SDS-PAGE showing the proteins in HeLa secretome obtained from the pericellular nanonets after 2 h of incubation of the precursors with the cells (2h_N) and collected by centrifugation of conditioned medium after 2 h of incubation (2h_CM). A piece of hydrogel (Gel) is loaded for SDS-PAGE and stained as the background. (B) Relative density of protein bands of 2h_N and 2h_CM in the two trials at molecular weight of 300, 160, 55, and 13 kDa. (C) According to protein LC-MS/MS, numbers of total peptide and unique peptide observed from proteins of the HeLa secretome obtained in 2h_N, 2h_CM, and the proteins in 2h_N no cells. (D) Numbers of total protein and identified protein (unique peptide number ≥2)[21,22] observed from protein LC-MS/MS in the HeLa secretome obtained in 2h_N and 2h_CM in two trials and proteins in 2h_N no cells. (E) Correlation of the unique peptide hits of proteins detected in 2h_N from the two trials. (F) Correlation of the unique peptide hits of proteins detected in 2h_CM from the two trials. (G) Comparison of identified proteins between 2h_N and 2h_CM (the listed proteins are identified in both trials of 2h_N or 2h_CM). HeLa secretome reported in literature[23] served as reference. (H) Analysis of the 122 proteins identified in both trials of 2h_N. The subcellular location information of the proteins was collected from UniProt. (I) Pie charts representing molecular functional profiles of the proteins in the secretome collected by pericellular nanonets from HeLa cells 2h_N and culture medium after 2h of incubation 2h_CM. The predicted secretory properties of the proteins were analyzed by Secretome 2.0 and the functional classification was analyzed by PANTHER.

To demonstrate that pericellular nanonets can rapidly collect secretory proteins, we conduct 3 experimental settings for the same incubation time 2h: first, pericellular nanonets collected from HeLa cells treated with **1**_**p**_ in complete culture medium (2h_N); second, the complete medium incubated with HeLa cells (2h_CM) for comparison; and finally, the complete medium treated with **1**_**p**_ and 0.1 U alkaline phosphatase without HeLa cells (2h_N no cells) as a control. Without additional processing, we directly apply electrophoresis to the samples. The self-assembled nanonets dissociate to monomeric **1** upon mixing with Laemmli loading buffer. Due to its small molecular weight (643 Da), **1** runs out of the gel and has little adverse effect on the staining or the further protein analysis of the gel. As shown in Fig 2A, despite being masked by serum proteins (mostly bovine albumins), silver staining of SDS-PAGE of both trials of 2h_N and 2h_CM reveals that 2h_N has darker stain than 2h_CM does. Image J[20] analysis shows that, in both trials, the bands at 300, 160, and 13 kDa all have obviously higher density in 2h_N than in 2h_CM, which illustrates that there are more proteins in these bands in 2h_N (Fig 2B). However, the band at 55 kDa, consisting mostly of bovine albumin from the culture medium, has higher density in 2h_CM than in 2h_N in trial I and has similar density in 2h_CM and 2h_N in trial II. These results suggest that the additional proteins collected by the nanonets are the secretory proteins from HeLa cells rather than serum proteins from the culture medium. As shown in Fig 2C, the mass spectrometry of the tandem protein shows more *total peptides* and *unique peptides* (peptide that exists only in one protein in human proteome) in 2h_N (1995 and 963 (trial I), 1952 and 990 (trial II)) than in 2h_CM (1401 and 603 (trial I), 1593 and 714 (trial II)) and 2h_N no cells (1209 and 784). Additionally, 2h_N also contains considerably more *total proteins* and *identified proteins* (the protein with 2 or more unique peptides) than 2h_CM and 2h_N no cells do (Fig 2D). These results agree with the observation in the silver stained SDS-PAGE that 2h N contains more proteins than CM collections.

While 2h_N trials have 161 and 181 identified proteins, only 144 proteins are identified in the 2h_N no cells trial, significantly less than previous trials with HeLa cells. The lack of identified proteins is expected because the absence of HeLa cells in this control experiment would not result in cancer secretome. The 2h_CM trials, on the other hand, have 128 and 108 identified proteins, which is slightly less than 2h_N no cells trial. One possible explanation for the unique protein difference between the nanonets and CM could be the potential concentrating effect of nanonets. It is possible that nanonets have certain affinity to low concentration proteins existing in the pericellular space and medium. This enrichment from nanonets may lead to some identified proteins in the results. In addition, we analyze the overlap of identified proteins between 2h_N no cells, 2h_N and 2h_CM. The average overlap between 2h_N no cells and 2h_N is 84 proteins, 30% (89 proteins, 30% for trail I and 79 proteins, 29% for trail II). The average overlap between 2h_N no cells and 2h CM is 63 proteins, 37% (60 proteins, 37% for trail I and 66 proteins, 36% for trail II). This additional control experiment proves that even though the nanonets may have a concentrating effect, the nanonets formed on the cancer cell surface still trap enough secretome to offset the possible influence from the medium enrichment.

To evaluate the pre-analytical variation between the two methods, nanonets and CM, we plot the identified proteins in the two trials (done by different operators) by the numbers of unique peptides (i.e., number of unique parent ions)[24] of the proteins. As shown in Fig 2E and 2F, the linear regression of the identified proteins in the two trials of 2h_N has a R^2^ value of 0.77, which is significantly higher than that of the two trials of 2h_CM (R^2^ value = 0.47), indicating the reproducibility between the two trials of 2h_N is much higher than that of 2h_CM. Moreover, the comparison of the unique peptide number of proteins observed in both 2h_N and 2h_CM indicates that most of these proteins have more unique peptides detected in 2h_N than in 2h_CM, thus confirming that pericellular nanonets collect more secretory proteins than CM does.

Furthermore, 67 of the total proteins identified in both trials of 2h_N (122 proteins) and in both trials of 2h_CM (81 proteins) (Fig 2G) are identical (Table A in S1 Table). By exploring the protein-protein interaction network[25] of these 67 proteins (S2 Fig), we find that most (50 proteins; 75%) are actins, serpins, collagens, tubulins and their interacting proteins. Despite being cytosolic in origin, actins, tubulins and serpins are commonly present in the secretome of human cells including cancer cells,[26,27] which agrees with the characteristic of unconventional protein secretion.[27] Besides these 67 proteins, 2h N contains 55 extra proteins (Table B in S1 Table), and 45 of them are reportedly associated with the progression of certain types of cancers. Among the 55 proteins observed only in 2h N, 50 of them have been observed in the secretome of other cancer cells. However, only 27 of the 55 proteins have been documented in the secretome of HeLa cells (obtained from ultra-filtrated CM of HeLa cells incubated with serum-free medium).[23] On the other hand, 2h_CM has only 14 additional proteins, other than the 67 shared proteins (Table C in S1 Table). These results indicate that 2h_N is more sensitive in collecting secretory proteins than 2h_CM. Interestingly, only 34.4% of the 122 identified proteins in 2h_N are secretory proteins and most of the rest are intracellular proteins, as categorized by UniProt.[28] However, feature-based prediction shows that over 60% of the proteins are secretory proteins, classical (by SignalP[29]) and non-classical (by SecretomP[30]) (Fig 2H). This data is similar to the evidence from the observation of body fluid whose secretome contains classical and non-classical secretory proteins, as well as intracellular proteins.[31] On the other hand, the protein profile of 2h_N no cells (Table I in S1 Table) only shows 35% of the total predicted secretory proteins. This result greatly corresponds to the fact that only culture medium proteins are collected without interference from HeLa cell secretome. We then used PANTHER[32] to analyze data from protein mass spectrometry of the samples of 2h_N and 2h_CM. While the percentages of most categories of the identified proteins are quite similar in the samples of 2h_N and 2h_CM, the secretome proteins collected by nanonets show higher percentages of proteins for catalytic, antioxidant, and binding activities(Fig 2I). The above results validate that the self-assembled nanonets act as a more accurate and sensitive method for collecting cancer secretome than CM.

### Total proteins from temporal profile of cancer secretome registered by nanonets

**Fig 3:**
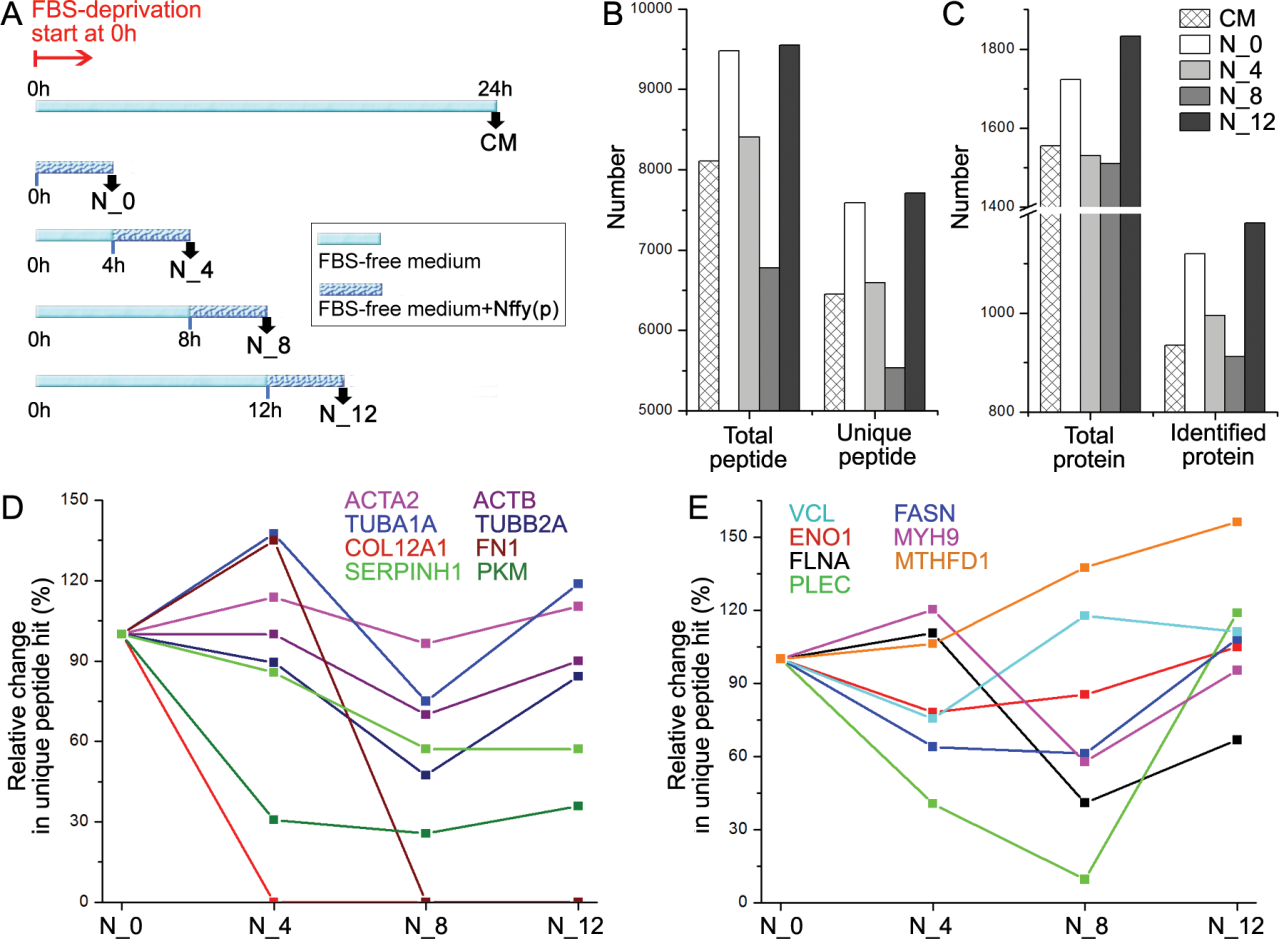
Pericellular nanonets register the dynamics of cancer secretome. (A) Scheme showing the collection of nanonets from HeLa cells exposed in FBS-free medium for different lengths of time. As a control, CM was collected from HeLa cells exposed in FBS-free medium for 24 h. (B) Number of total peptide and unique peptides observed in N_0, N_4, N_8, N_12, and CM by protein mass spectrometry. (C) Number of total protein and unique protein (unique peptide number ≥2)[34,35] observed in N_0, N_4, N_8, N_12, and CM by protein LC-MS/MS. (D) The change in unique peptide number of several essential proteins in secretome observed in the nanonets. (E) The change in unique peptide number of several representative proteins (collected in the nanonets) that have high unique peptide hits observed in MS protein profiling.

The short incubation time enables the pericellular nanonets to register the dynamics of cancer secretome. As shown in Fig 3A, incubating the HeLa cells in FBS-free medium for different lengths of time and then changing the medium to FBS-free medium containing **1**_**p**_ for an additional 4 h of incubation produces nanonet-collected secretome of HeLa cells. The control experiment uses CM to collect the secretome of HeLa cells incubated in FBS-free medium for 24 h.[33] According to protein mass spectrometry analysis of these samples (Fig 3B), the amounts of total peptides and unique peptides decrease from the N_0 sample to the N_8 sample before the trend reverses for the N_12 sample, which has a slightly higher amount of peptides than the N_0 sample. Although CM provides the useful accumulative data of HeLa secretome after the standard 24 h incubation, it is obviously unable to capture the dynamic change of the amount of the proteins in the secretome during 24 h of incubation.

### Categories of the proteins remain globally balanced

**Fig 4:**
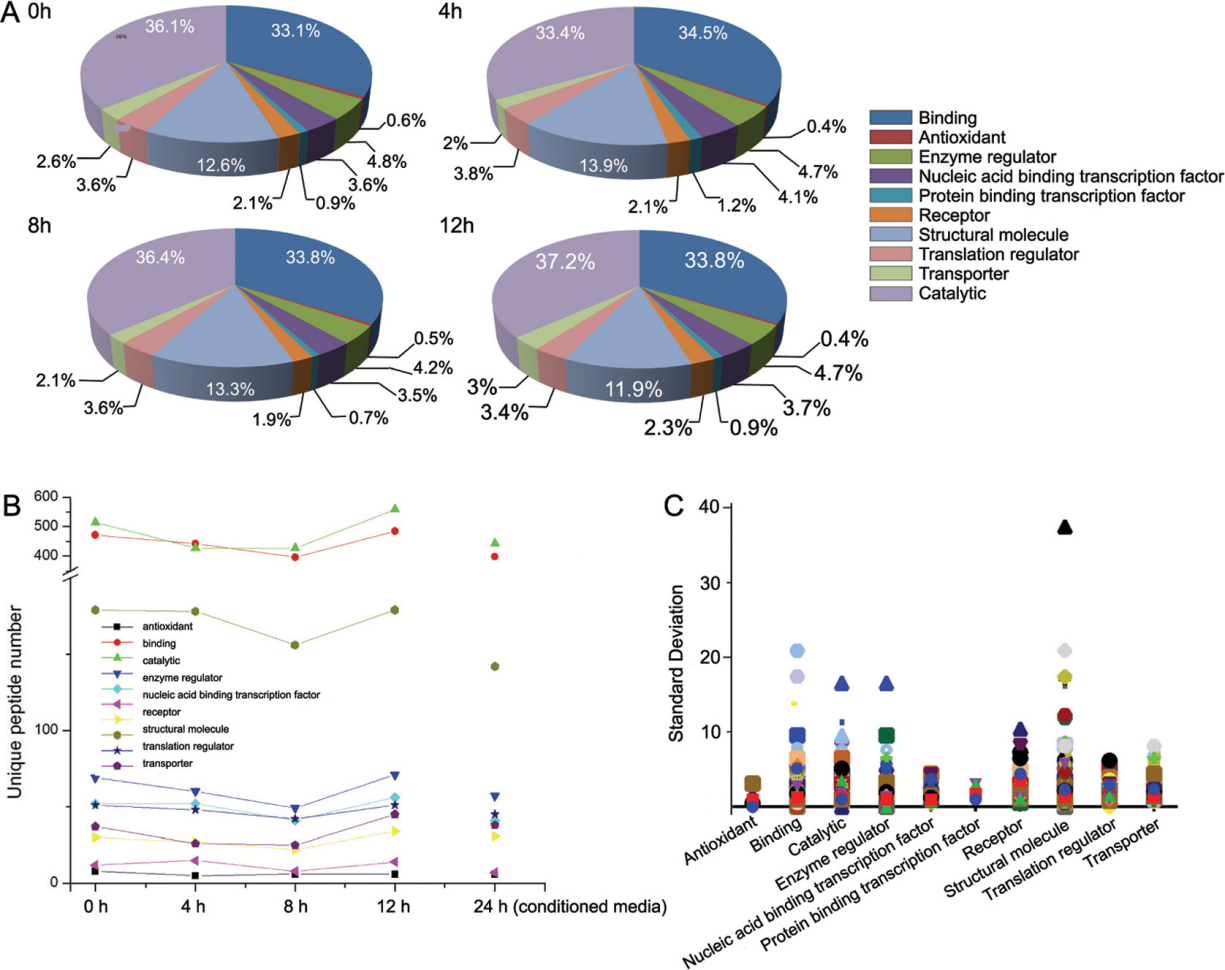
The temporal profiles of categorized proteins in the cancer secretome. (A) Temporal pie charts represent molecular functional profiles of cancer secretome collected after the FBS-deprivation for different lengths of time: 0 (N_0), 4 (N_4), 8 (N_8), and 12 h (N_12). (B) The plot represents temporal behaviors of different categories of cancer secretome based on molecule functions, during the FBS-deprivation for different lengths of time (0, 4, 8 and 12 h) and incubation in conditioned media for 24 h. (C) The plot illustrates the fluctuating range (shown as standard deviation) of the temporal behavior for categorized secretomes.

Although certain individual proteins in the secretomes exhibit considerable variation, the functional categories of the proteins in the secretome remain rather constant. We use PANTHER to analyze data from the LC-MS/MS of these samples with pre-incubation times of 0 to 12 h (Fig 4A, Table H in S1 Table). The percentages of each category of identified proteins remain almost constant. Eight of ten (Fig 4B) categories of the secretomes show the same trend in temporal changes—the amount of proteins initially decrease followed by increasing later on. The activity of antioxidant and protein binding transcription factors show different responses to the deprivation of nutrients (i.e., FBS free medium): antioxidant proteins increase after a short interval (4 h) of nutrient deprivation; protein binding transcription factors increase after a median interval (8 h) of nutrient deprivation. Interestingly, these two types of activities share comparatively low baselines in terms of their small amount of proteins. Because the general trend of the composition of proteins is to remain constant to FBS-deprivation, the large variation of individual categories of small amounts of proteins is unlikely to be caused by attempts to maintain proteostasis.

To better understand the secretome temporal profiles during FBS-deprivation, we also include the result of 24 h of incubation in conditioned media (CM) as the reference (Fig 4B). As shown in Fig 4B, the amount of proteins in CM is close to the average amount of proteins in the secretomes during temporal changes (from 0 to 12 h). This result implies that the global cellular activities are likely to remain balanced during the FBS deprivation. In addition, these results indicate that 4 h of incubation with the nanonets leads to minimal perturbation of the secretome. To understand the temporal change of the secretome in a more precise manner, we also calculate the relative amounts of proteins (S3–S7 Figs) and standard deviation values in each category of proteins. As shown in Fig 4C, the distribution of standard deviations of the temporal changes of each category of protein differs between protein categories. For example, while the standard deviation value of the secreted proteins in the category of structural molecular activity and binding is larger than 20, the standard deviation value of the secreted proteins in the category of antioxidant and protein binding transcription factor activity is lower than 5. This distinction in distribution indicates that the extent of the responses to FBS-deprivation varies between each category of secretory proteins. These results, thus, provide a useful insight to the behavior of cancer cells under nutrient deprivation.

### Relative changes of proteins in each functional category

**Fig 5:**
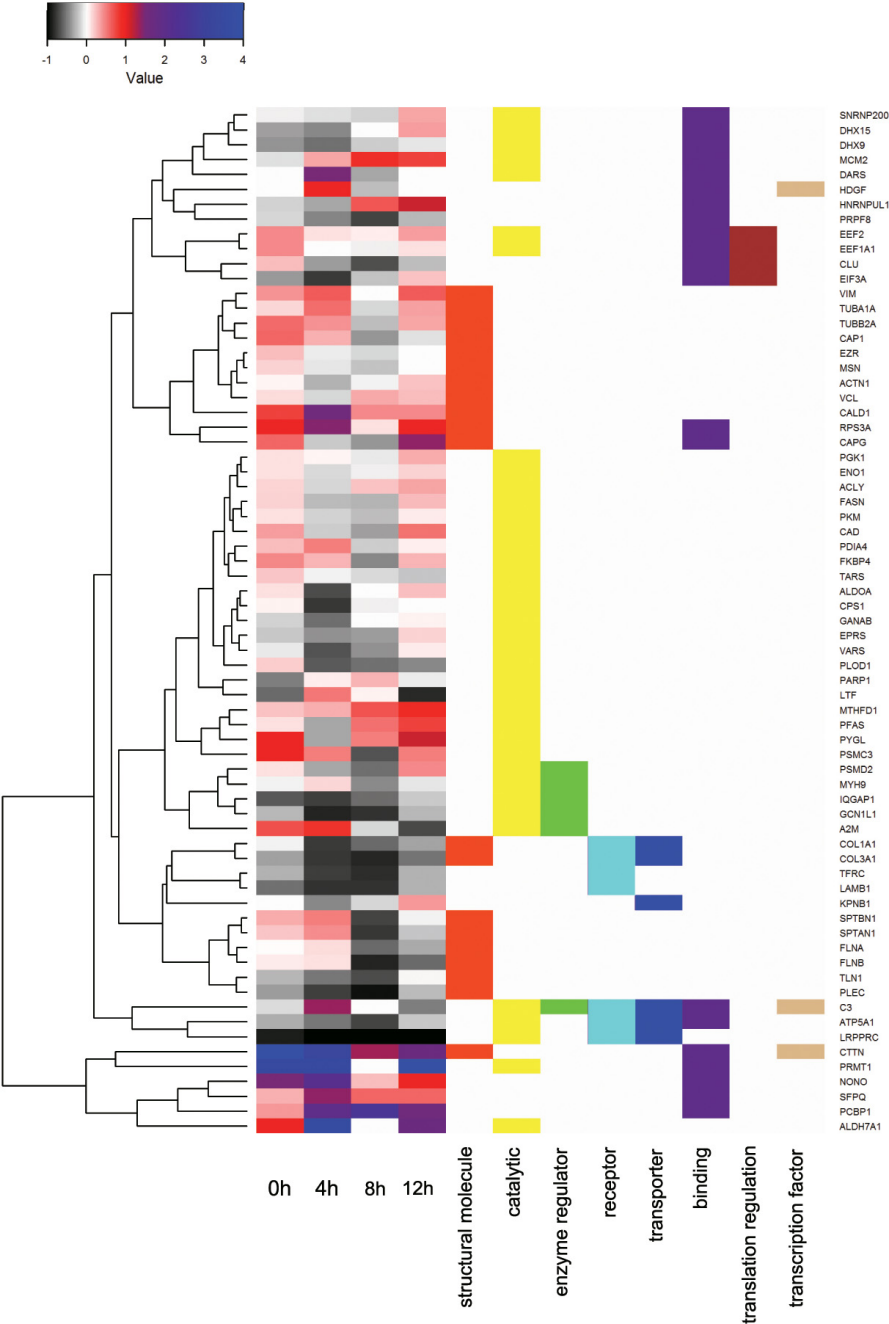
Heat map representation shows the relative temporal change and their functional categories based on GO database annotation for 69 proteins selected by sorting standard deviation from largest to smallest.

The relative change of the amount of proteins, on the other hand, shows an unbiased changing profile of secretome by using the definite difference between each time point divided by the results of a control group (24 h of incubation in FBS-free medium) throughout the experimental time period. The result of relative change (RC value) for each secretome is calculated using the following formula: $RC\;value=\frac{\left(unique\;peptide\;number\;of\;each\;time\;point-unique\;peptide\;number\;of\;control\right)}{unique\;peptide\;number\;of\;control}$. The comparison of temporal change pattern based on RC value for 69 selected proteins with significantly changing profiles is illustrated in Fig 5. Since certain proteins may belong to more than one category, information on the functional category of each protein is included in the heat map. The overall range of RC values (from -86% to 100%) reflect the general trends of stable secretion of cancer cell during the stimulus of FBS-deprivation. Several proteins exhibit a large RC value (PCBP1, ALDH7A1, PRMT1, CALD1, etc.). This phenomenon is likely due to the small number of detected unique peptides in the control. This result further indicates that using nanonets in place of CM is a more powerful and suitable method for collecting cancer secretome.

From 0 to 4 h, 42 out of 69 proteins (shown in color red to blue) exhibit positive RC values (Fig 5), suggesting an increase in the amount of the protein in response to a lack of nutrients. After another 4 h, the general trend becomes a decrease of secretion, as evidenced by negative RC values of 38 of 69 proteins (shown in color grey to black). During the listed time periods of 8–12 h and 12–16 h, the secretion also insinuates a similar decreasing pattern since the amount of secretion exhibits negative RC values for 46 and 39 out of 69 proteins, respectively, throughout these two periods. A categorized analysis (Fig 5) of cancer secretome reveals that most of the proteins within the same functional category rarely share a common trend of temporal profiles of secretion upon stimulation. However, for the multifunctional proteins (i.e., belong to different functional categories), their temporal profiles usually exhibit similar trends over the entire stimulation time. For example, the proteins grouped into functional categories of both binding and catalytic activity, such as MCM2, DHX9, DHX15, SNRNP200, all show a decrease in abundance for the first 4 h and subsequent increase for the remainder of FBS-deprivation; but other proteins in only one of these two categories (either binding or catalytic category) hardly show common features. This character is apparently unique for “multifunctional” proteins. For example, GCN1L1, PSMD2, and IQGAP1, which belong to both catalytic and enzymatic activities, exhibit the same amount of secretion increase for the initial 4 h, follow by decrease. This phenomenon was unknown previously, and it certainly warrants further studies.

### The dynamic change of certain specific proteins

Since dynamic changes of the secretome of cancer cells have been previously unattainable, although containing highly important information, we analyze changes in abundance of several essential proteins in the samples from N_0 to N_12. Comparing the numbers of unique peptide hits of these proteins helps to establish their temporal profiles (Fig 3D and 3E). As shown in Fig 3D, the amount of alpha-actin 2 (ACTA2) and beta-actin (ACTB) exhibit little change over time (from 0–4 h (N_0) to 12–16 h (N_12)) and the amount of alpha-tubulin 1A (TUBA1A) and beta-tubulin 2A (TUBB2A) only change slightly over time, agreeing with the observation that actins and tubulins have a constant presence in the secretome of mammalian cells.[26,27] On the contrary, the amounts of both collagen alpha-1(XII) (COL12A1) and fibronectin (FN1) decrease drastically, that is, the collagen alpha-1(XII) disappears from N_4 to N_12, and the fibronectin is absent in N_8 and N_12. The amount of two enzymes, serpin H1(SERPINH1) and pyruvate kinase (PKM), also decrease significantly from N_0 to N_12. These results suggest that, to cope with the starvation induced by FBS-deprivation, the HeLa cells significantly decrease or possibly reabsorb the extracellular matrix proteins and the secreted enzymes for maintaining proteostasis.[36] We also examined several proteins with high unique peptide hits (Fig 3E). Notably, the amount of plectin (PLEC) undergoes the largest change from N_0 to N_12. While the decrease of plectin from N_0 to N_8 by the HeLa cells is likely for the purpose of maintaining proteostasis, the sudden increase of plectin in N_12 indicates an alleviated release of exosome,[37] agreeing the starvation behavior cancer cells that attempt to manipulate tumor microenvironments (i.e., stimulate stromal and endothelial cells) to aid their progression.[38,39] There is another interesting observation: while the amount of all other proteins decrease in a certain degree in at least one sample of nanonets, the amount of pyruvate kinase (PKM) gradually increases over time. As pyruvate kinase is the rate-limiting enzyme in glycolysis,[40,41] the increased level of pyruvate kinase is consistent with the need for increased ATP production by the cancer cells.

### Nanonets reveal the transient change of cancer secretome after changing stimuli

**Fig 6:**
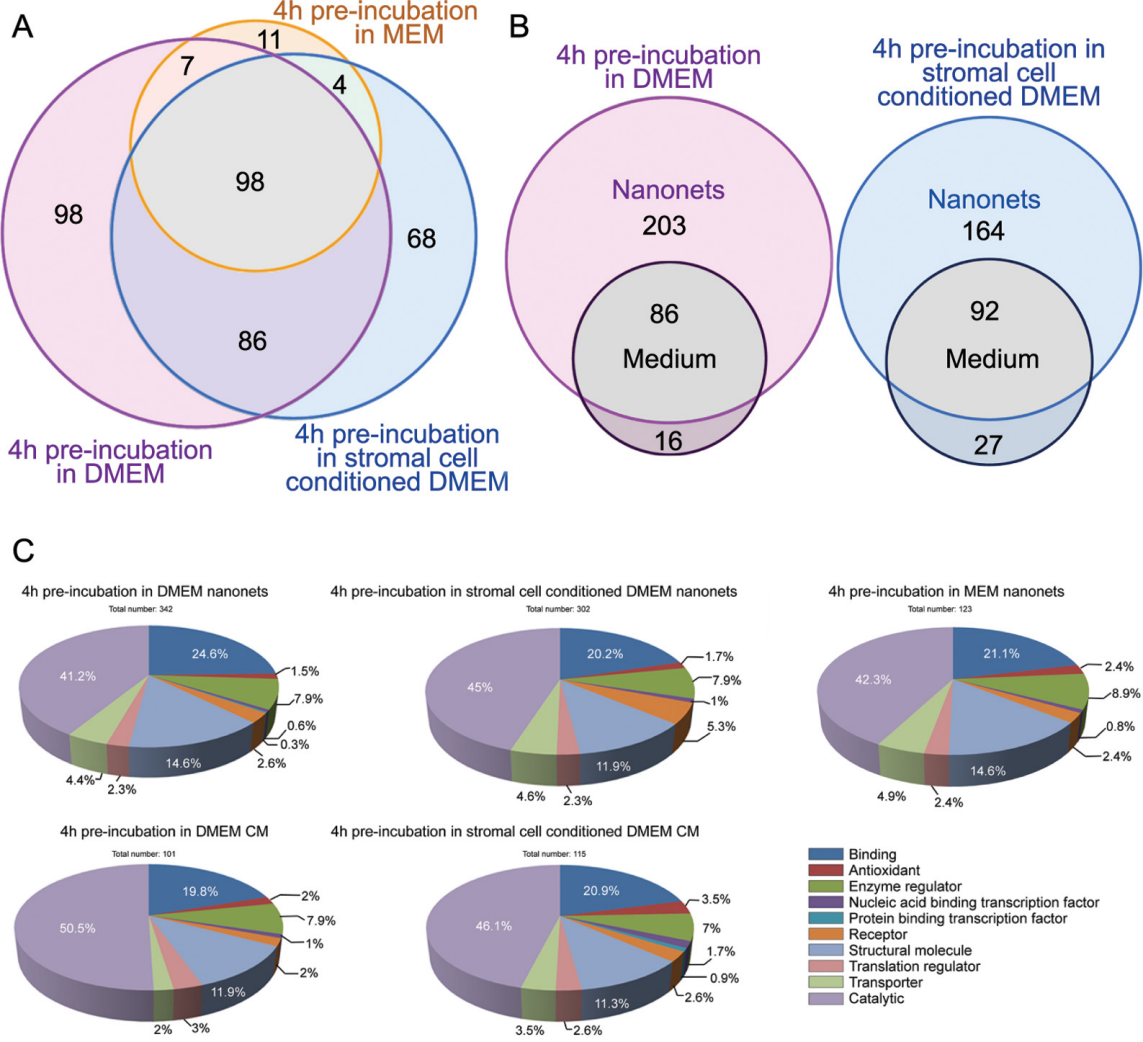
Nanonets collect different secretory proteins from HeLa cells pretreated by different culture media. (A) 4 h pre-incubation in DMEM and 4 h pre-incubation in stromal cell conditioned DMEM lead to increase of secretory proteins of HeLa cells, compared with HeLa cells incubated in complete MEM. (B) 24 h CM collect significantly fewer secretory proteins than do the pericellular nanonets. (C) Pie charts represent molecular functional profiles of cancer secretome collected upon changing media.

To demonstrate that the nanonets are also able to register the changes in cancer secretome triggered by other types of stimulation, we pre-treat the HeLa cells in different culture media for 4 h, and then use the nanonets to collect the secretome. Fig 6A shows the comparison of proteins in the secretome collected by the pericellular nanonets from HeLa cells pre-incubated with DMEM for 4 h (Table D in S1 Table), HeLa cells pre-incubated with a stromal cell (HS-5) conditioned DMEM for 4 h (Table E in S1 Table), and HeLa cells incubated only in complete MEM. Substantially more proteins are collected by the nanonets from the pre-treated HeLa cells than from HeLa cells without pre-treatment. According to the results from protein profiles, the proteins observed from the HeLa cells pre-incubated with DMEM differ considerably from the proteins observed from the HeLa cells pre-incubated with stromal cell conditioned DMEM. The 289 proteins captured by nanonets from HeLa cells with unconditioned DMEM share an overlay of 184 proteins with the 256 captured by nanonets from HeLa cells with HS-5 conditioned DMEM. This result suggests that the HeLa cells secrete different proteins to respond to different stimuli. However, some proteins exhibit high abundances in both cases, their unique and total peptide numbers can be similar. For example, ENO1 and PKM show the largest unique and total peptide hits in both secretome profiles, which are 29/93 and 22/57 in untreated DMEM, 29/89 and 25/53 in HS-5 stromal cells conditioned DMEM. This similarity also applies to some other proteins with significantly large unique and total peptide hits, such as ACTA2, ACTN4, GAPDA, HSPA5, HSPA8, TUBA1A and TUBB2A. On the other hand, the change of media induces approximately double or half of the secretion of some proteins. For example, proteins CPS1, VCL and ATIC have twice the amount of unique and total peptide hits with the unconditioned DMEM compared to that with HS-5 conditioned media. Proteins A2M, AHCY, and EEF1A1 behave oppositely, having about half the amount of unique and total peptide hits in the unconditioned DMEM compared to HS-5 conditioned media.

Just like the previous demonstration (Fig 3B) of a more complete secretome profile captured by pericellular nanonets than CM, in the experiment of changing stimuli, the pericellular nanonets obtained in a short time span, 4 hours, also clearly collect more secretory proteins than CM does over 24 h of incubation after pre-treatment (Tables F and G in S1 Table). The fact that CM gathers fewer proteins than pericellular nanonets confirms that the increase of protein secretion after the stimulation is a rather transient process, agreeing with the dynamics of cancer cells.

**Fig 7:**
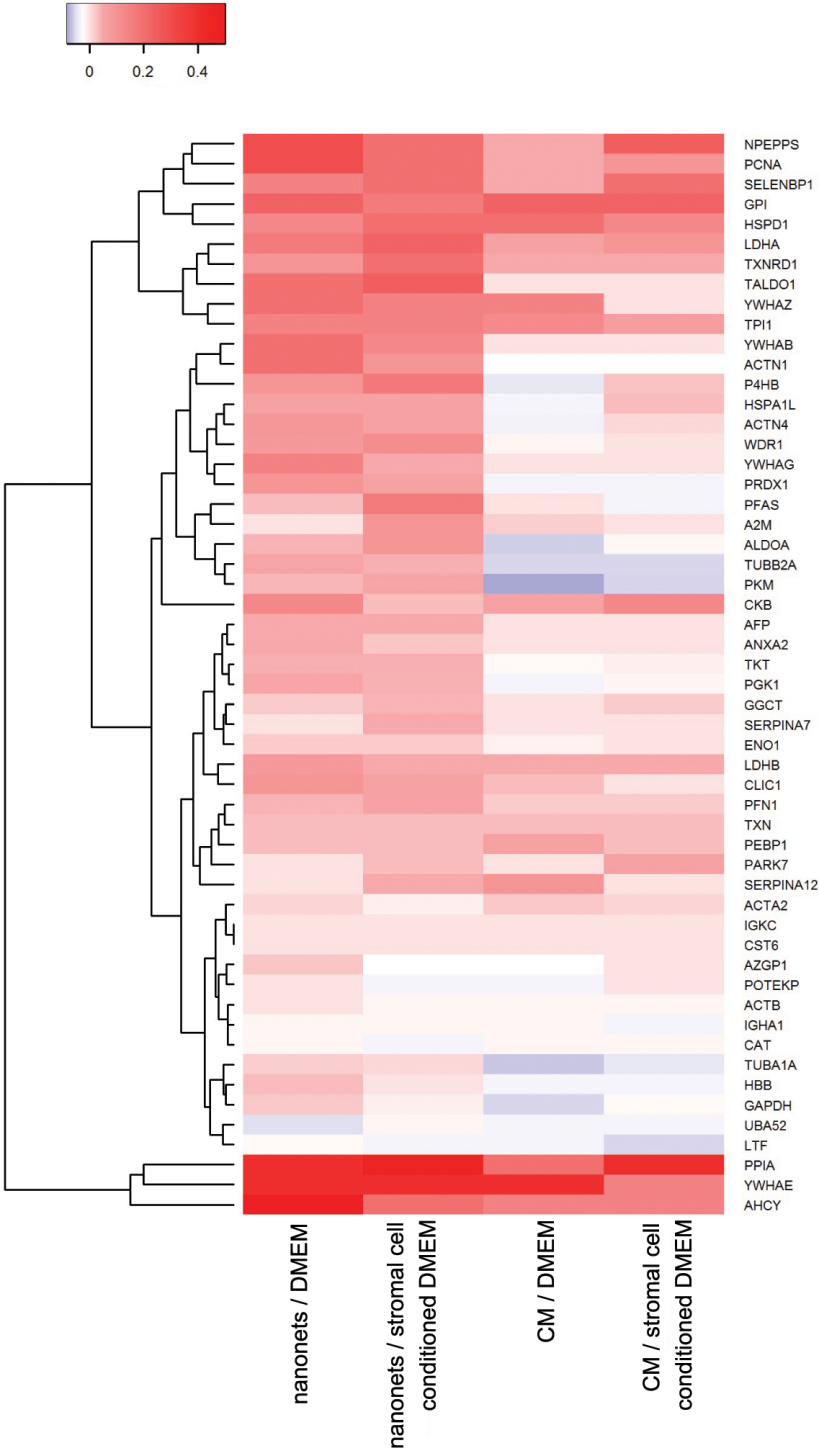
Heat map representation shows the relative change of 54 proteins selected by sorting standard deviation largest to smallest.

The differences in the individual secretome profiles between changed media (DMEM and stromal cell conditioned DMEM) and the control group (MEM as reference) are illustrated in a heat map (Fig 7). After changing the media from MEM to DMEM, HeLa cells show mostly increased secretion, represented by positive values (red) of relative change (RC) for the proteins that have increased amount of unique peptide hits compared to the control group in MEM. As expected, nearly all the negative RC values are from the secretome results of CM collection, which also validates our previous observation that nanonets provide a higher yield of secretome than CM. This increased yield of collecting secretion is consistent because RC value ranges from 0 to less than 0.5, with most of RC values below 0.2. This small range of RC indicates that the additional amino acids and vitamins from DMEM (compared to MEM) have a limited influence and apparently only induce small changes to HeLa cell secretion.

The first two columns (in Fig 7) document and compare the secretomes captured by the nanonets after incubation of HeLa cells in two different media: DMEM and HS-5 conditioned DMEM. It is known that HS-5 conditioned media promotes differentiation[42] of monocytes to dendritic cells, and may provide additional cytokines and interleukins[43] that help cell growth. Notably, some proteins show relatively different profiles. For instance, the amount of CKB (a cytoplasmic enzyme involved in energy homeostasis[44]) is lower in HS-5 conditioned medium (unique peptide hits = 5) than unconditioned DMEM (unique peptide hits = 7). This difference, however, is unlikely to reflect the difference in cell response because the sum of the unique peptide hits of CKB from nanonets and CM indicates that the total amount of peptide hits stay almost the same (12 in HS-5 conditioned media and 11 in untreated DMEM). Thus, in the conditioned media, CKB tends to accumulate more in CM than in nanonets, suggesting that the secretion of CKB may be stable upon media change, but abundance of CKB in CM is greater than that in nanonets with HS-5 conditioned medium.

The above results not only reveal that HeLa cells regulate the secretome to cope with changes of their environment,[45] but also imply that the use of pericellular nanonets may ultimately lead to an effective method to monitor the transient changes and the dynamics of cancer secretome in tumor microenvironments[46] and identify proteins that may be abnormally secreted, shed, or overexpressed upon stimulation.

## Discussion

The results in this work demonstrate that the use of the biochemical catalysis (i.e., enzymatic dephosphorylation) of cancer cells to ensure the collection of secretome occurs in-situ (i.e., pericellular space) enables a rapid, direct, and comprehensive collection of cancer secretome. The high yield of proteins collected by the nanonets (in most cases), the observation of autocrine AFP[47] (a potential cancer biomarker,[19,48] that has not been documented in HeLa secretome[23]) in relative short time (4 h), and the observation of exosomal protein, plectin, all together suggest the high sensitivity of using nanonets for profiling secretory proteins. Yet, compared to other collecting methods, protein mass spectrometry identifies a little less secretory proteins from the nanonets (1,120 identified protein) than that from ultrafiltration concentrated CM (1,223 identified protein),[23] with both the nanonets and the CM obtained from the HeLa cells in FBS-free medium. Although these two methods collect similar amounts of the secretome, the use of nanonets is more effective than CM, in terms of speed and simplicity. Moreover, the facile collecting procedure based on nanonets eliminates further purification or concentration process. The collecting procedure of nanonets, which significantly reduces pre-analytical variation between trials and improves sensitivity, suggest that it is not only suitable to combine pericellular nanonets with other proteomic techniques, such as SILAC,[49] for accurate and comprehensive mapping of cancer secretome, but also may evolve into a low-cost and rapid diagnostic method for developing regions.[50] The short incubation time also allows the nanonets to capture the temporal changes in the cancer secretome in response to stimulation, which may reveal new insights on cancer drug resistance. In addition, the spatiotemporal control over the formation of the nanonets may ultimately lead to single cell analysis[51,52] of secretome in tumor microenvironments.

## Supporting Information

S1 Fig Optimal incubation time with Napffy(p) is between 2h to 6h. (A) Western blot showing the amount of tubulins in CM collected after 24h of incubation or pericellular nanonets collected after 3 to 9h of incubation. Bar graph shows the relative density of the tubulin bands. Both CM and nanonets were collected in FBS-free MEM. (B) Viability of cells after cold shock and nanonet collection. The cells were incubated with Napffy(p) for 4h in FBS-free MEM. The viability of the cells was tested in two trials each with three repeats.
(TIF)

S2 Fig Networks of the proteins identified in both trials of 2h N and 2h CM. Actins, serpins, GAPDH, collagens, and their directly interacting proteins are marked by red dots.
(TIF)

S3 Fig The lots represent relative temporal change of secretome amount during the FBS-deprivation for different lengths of time (N_0, N_4, N_8 and N_12) in (A) ligase activity, (B) transferase activity, (C) nucleic activity and (D) oxidoreductase activity.
(TIF)

S4 Fig The plots represent temporal relative change of secretome amount during FBS-deprivation for different lengths of time (N_0, N_4, N_8 and N_12) in (A) lyase activity, (B) hydrolase activity, (C) receptor activity and (D) calcium-dependent phospholipid activity.
(TIF)

S5 Fig The plots represent relative temporal change of secretome amount during FBS-deprivation for different lengths of time (N_0, N_4, N_8 and N_12) in (A) nucleic acid binding transcription factor, (B) protein binding, (C) translation regulation activity and (D) structural molecule activity.
(TIF)

S6 Fig The plots represent relative temporal change of secretome amount during FBS-deprivation for different lengths of time (N_0, N_4, N_8 and N_12) in (A) antioxidant activity, (B) calcium-dependent phospholipid binding, (C) isomerase activity and (D) helicase activity.
(TIF)

S7 Fig The plots represent relative temporal change of secretome amount during FBS-deprivation for different lengths of time (N_0, N_4, N_8 and N_12) in (A) calcium ion binding, (B) enzyme regulator activity, (C) chromatin binding and (D) deaminase activity.
(TIF)

S1 File Supplementary Information. (DOCX)

S1 Table Lists of identified proteins in different trials. (A) List of 67 proteins identified in both trials of 2h_N and 2h_CM. (B) List of 55 other proteins identified in both trials of 2h_N. (C) List of 14 other proteins identified in both trials of 2h_CM. (D) List of proteins identified in 4h nanonets from HeLa cells pre-treated by DMEM for 4h. (E) List of proteins identified in 4h nanonets from HeLa cells pre-treated by stromal cell conditioned DMEM for 4h. (F) List of proteins identified in 24h CM from HeLa cells pre-treated by DMEM for 4h. (G) List of proteins identified in 24h CM from HeLa cells pre-treated by stromal cell conditioned DMEM for 4h. (H) List of proteins identified in the temporal profiles (N_0, N_4, N_8, N_12) with molecular function and unique peptide number. (I) MS data for additional control experiment 2h_CM no cells.
(XLSX)

S2 Table Lists of identified proteins in temporal profiles (Raw MS Data). (A) List of proteins identified in N_0. (B) List of proteins identified in N_4. (C) List of proteins identified in N_8. (D) List of proteins identified in N_12. (E) List of proteins identified in CM_24.
(XLSX)

S1 Video Demonstration of the collection of nanonets. (MP4)

S2 Video Demonstration of the collection of CM. (MP4)
