# Supplementary figures and images for: Nanonets Collect Cancer Secretome from Pericellular Space
Source: PLoS One. 2016 Apr 21;11(4):e0154126. doi: 10.1371/journal.pone.0154126 (PMC4839576; doi:10.1371/journal.pone.0154126)

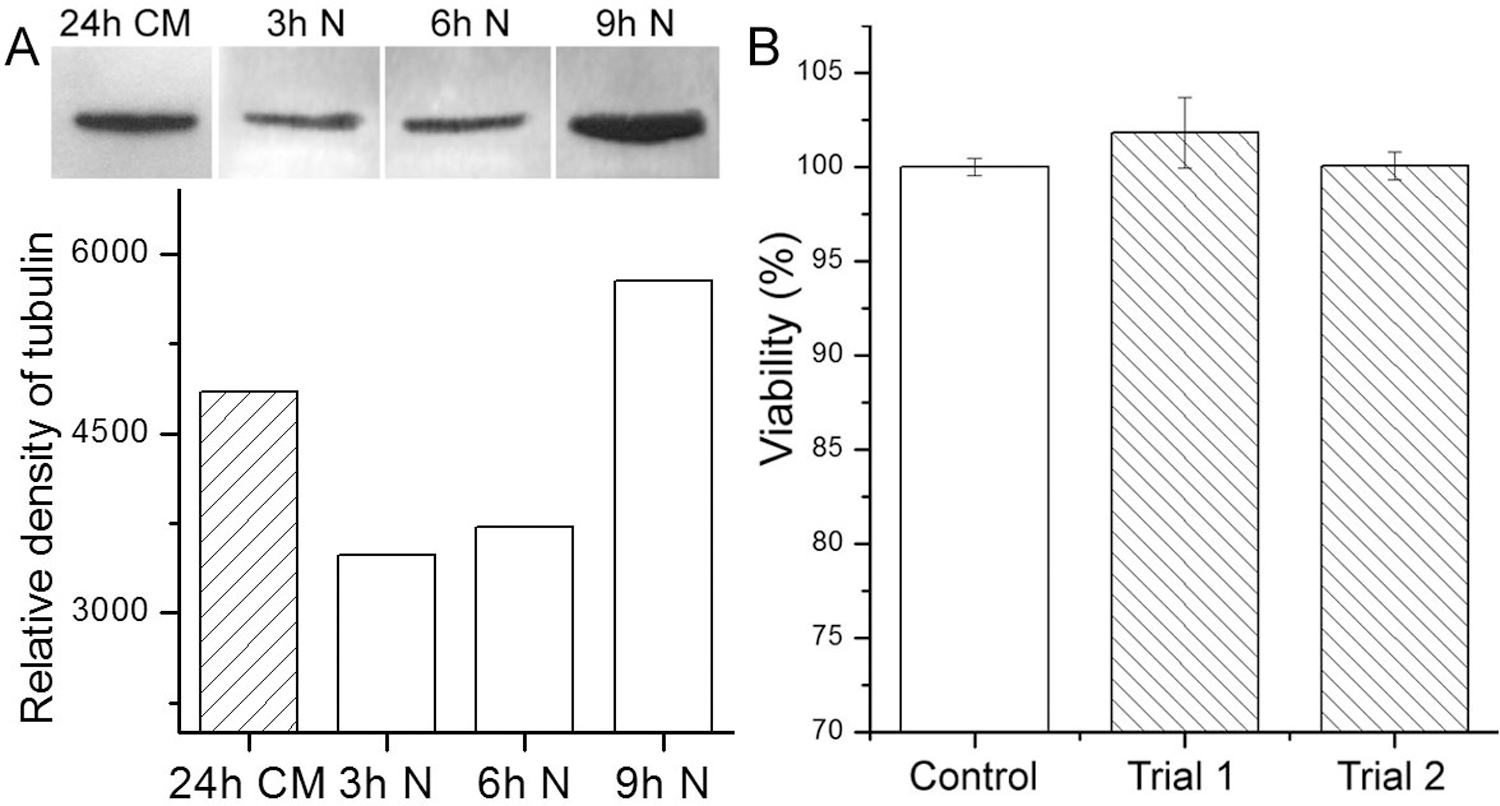

Supplement: S1 Fig — (A) Western blot showing the amount of tubulins in CM collected after 24h of incubation or pericellular nanonets collected after 3 to 9h of incubation. Bar graph shows the relative density of the tubulin bands. Both CM and nanonets were collected in FBS-free MEM. (B) Viability of cells after cold shock and nanonet collection. The cells were incubated with Napffy(p) for 4h in FBS-free MEM. The viability of the cells was tested in two trials each with three repeats. (TIF) [file pone.0154126.s001.tif]

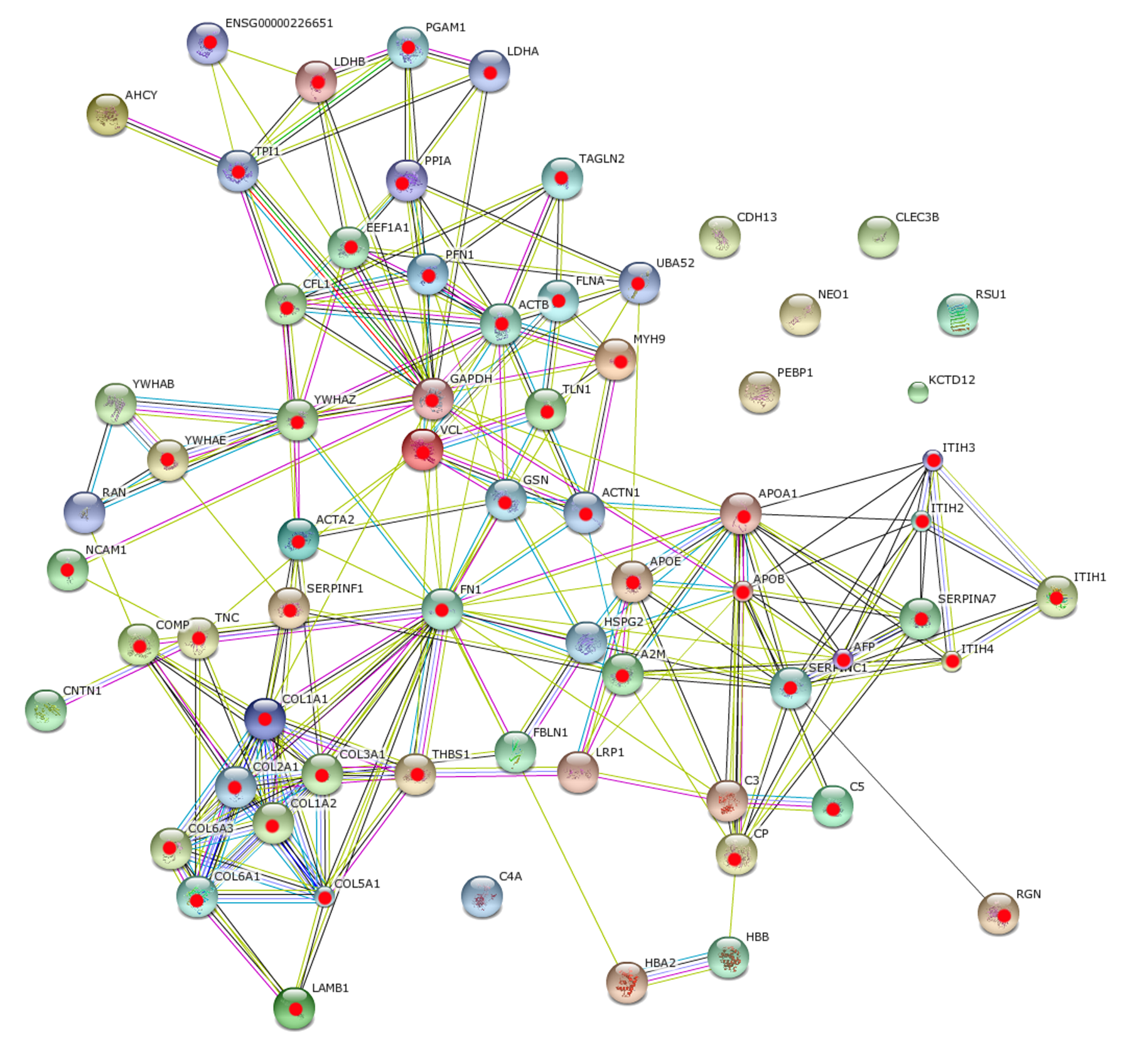

Supplement: S2 Fig — Actins, serpins, GAPDH, collagens, and their directly interacting proteins are marked by red dots. (TIF) [file pone.0154126.s002.tif]

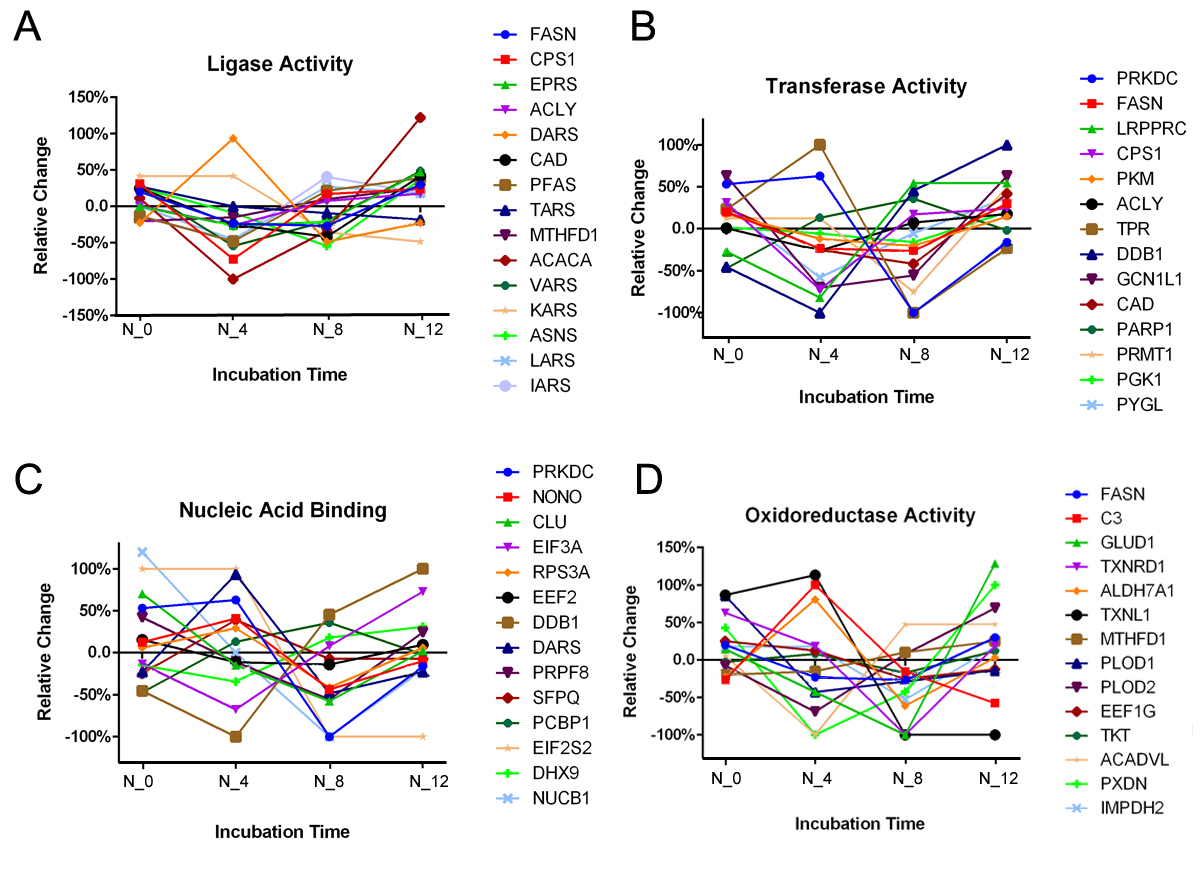

Supplement: S3 Fig — The lots represent relative temporal change of secretome amount during the FBS-deprivation for different lengths of time (N_0, N_4, N_8 and N_12) in (A) ligase activity, (B) transferase activity, (C) nucleic activity and (D) oxidoreductase activity. (TIF) [file pone.0154126.s003.tif]

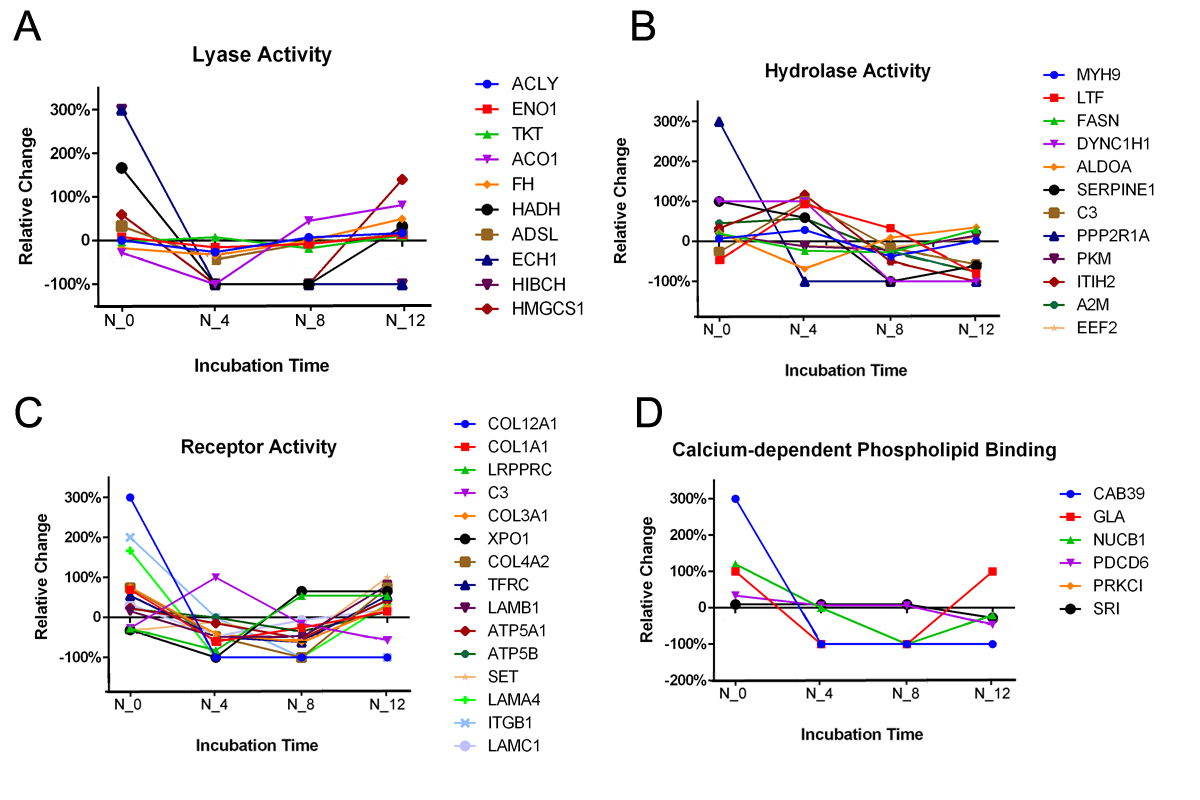

Supplement: S4 Fig — The plots represent temporal relative change of secretome amount during FBS-deprivation for different lengths of time (N_0, N_4, N_8 and N_12) in (A) lyase activity, (B) hydrolase activity, (C) receptor activity and (D) calcium-dependent phospholipid activity. (TIF) [file pone.0154126.s004.tif]

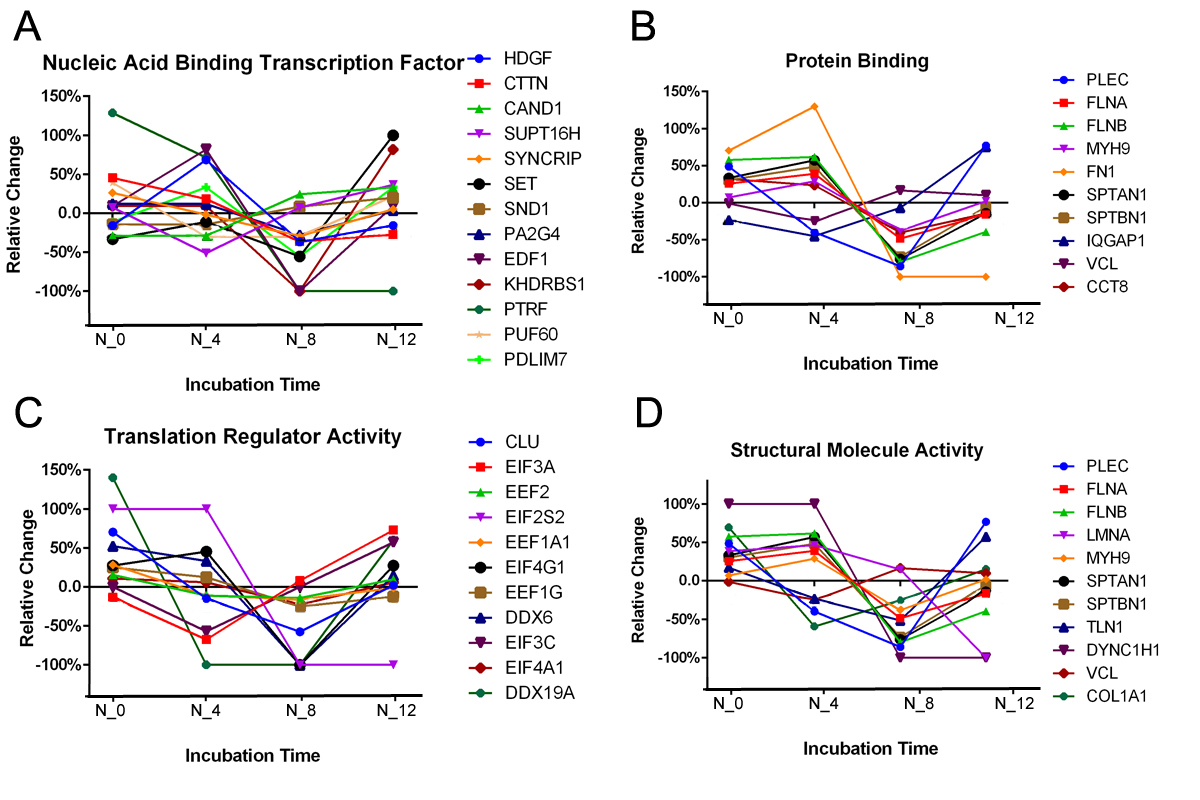

Supplement: S5 Fig — The plots represent relative temporal change of secretome amount during FBS-deprivation for different lengths of time (N_0, N_4, N_8 and N_12) in (A) nucleic acid binding transcription factor, (B) protein binding, (C) translation regulation activity and (D) structural molecule activity. (TIF) [file pone.0154126.s005.tif]

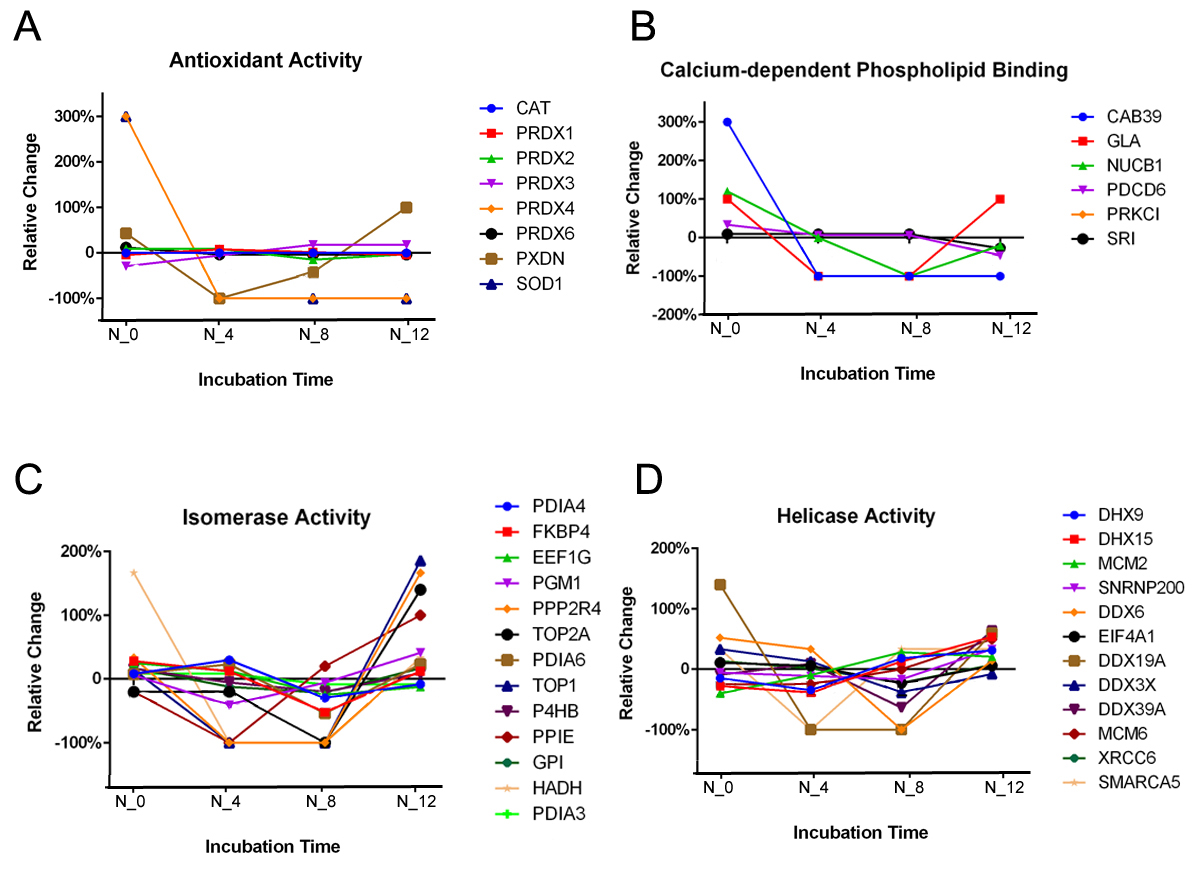

Supplement: S6 Fig — The plots represent relative temporal change of secretome amount during FBS-deprivation for different lengths of time (N_0, N_4, N_8 and N_12) in (A) antioxidant activity, (B) calcium-dependent phospholipid binding, (C) isomerase activity and (D) helicase activity. (TIF) [file pone.0154126.s006.tif]

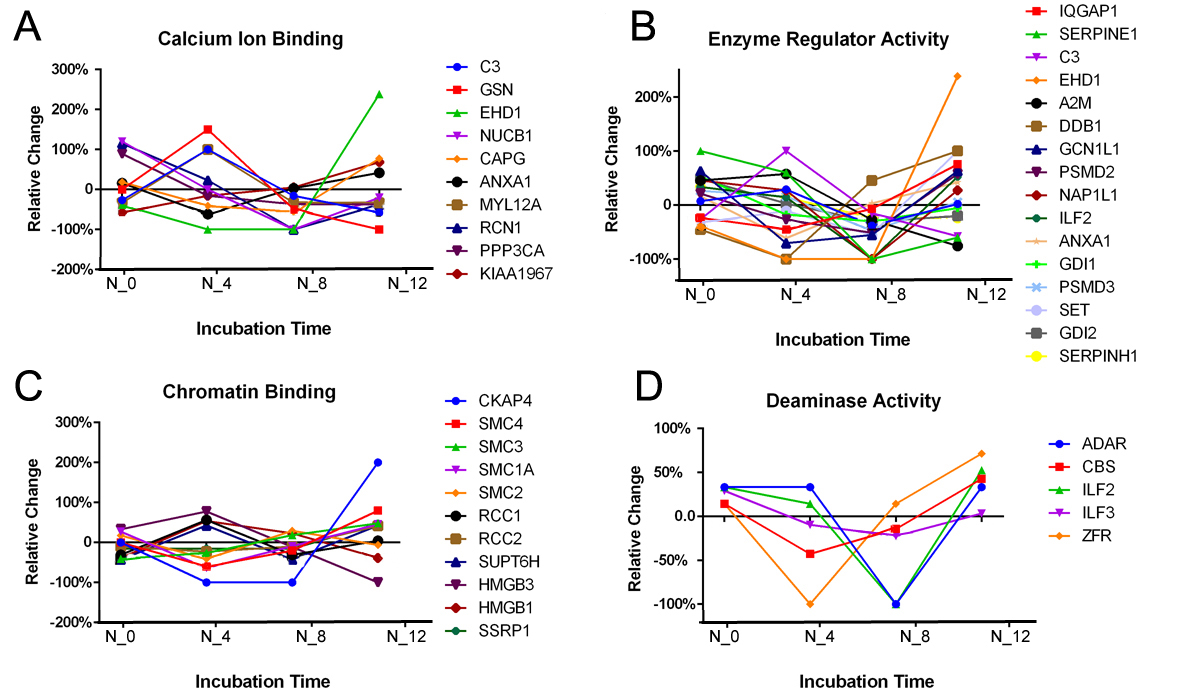

Supplement: S7 Fig — The plots represent relative temporal change of secretome amount during FBS-deprivation for different lengths of time (N_0, N_4, N_8 and N_12) in (A) calcium ion binding, (B) enzyme regulator activity, (C) chromatin binding and (D) deaminase activity. (TIF) [file pone.0154126.s007.tif]
