# Supplementary material for: Nanonets Collect Cancer Secretome from Pericellular Space
Source: PLoS One. 2016 Apr 21;11(4):e0154126. doi: 10.1371/journal.pone.0154126 (PMC4839576; doi:10.1371/journal.pone.0154126)
Supplement: S1 File — (DOCX) [file pone.0154126.s008.docx]

Supplementary Information for:

**Self-assembled Pericellular Nanonets to Reveal the Temporal Profile of Cancer Secretome**

Rong Zhou, Yi Kuang, Jie Zhou, Xuewen Du, Jie Li, Junfeng Shi and Bing Xu*

Department of Chemistry, Brandeis University, Waltham, MA 02454, USA.

Correspondence to: bxu@brandeis.edu

**This file includes:**

Supplementary Methods

**Supplementary Methods*:***

*FBS deprivation and the collection of nanonets*: 3×10^5^ of HeLa cells in 2 mL of complete MEM medium were seeded into a 35 mm Petri dish. After 24h incubation, the medium was removed, and the cells were washed 3 times with 1 mL FBS-free MEM medium. For the collection of CM, the cells were applied with 1 mL of FBS-free MEM medium and were incubated at 37˚C for 24h. 100 µL of the medium were collected after incubation. For collection of nanonets, the cells were applied with 1 mL of FBS-free MEM medium and incubated at 37˚C for 0 (add and immediately remove), 4, 8, or 12h. After the incubation, the cells were washed 3 times with 1 mL FBS-free MEM medium and then applied with 3 times with 1 mL FBS-free MEM medium containing Nffy(p) at 0.4 mg/mL (diluted from a 20X stock solution of the Nffy(p) in PBS buffer). After 4h of incubation at 37˚C, the nanonets/hydrogels were collected as mentioned above.

*Stromal cell conditioned medium*: 6×10^5^ of HS-5 cells in 5mL of complete DMEM medium were seeded in to a 60 mm Petri dish. After 24h incubation at 37˚C, the medium were replaced by 5mL of complete DMEM medium. The cells were incubated for 24 h at 37˚C. The medium were removed and centrifuged for 1 min to remove dead cells. The suspension of the conditioned medium was snap freeze in liquid N_2_ and stocked at -80˚C until use.

*Pre-treatment of incubation with different medium*: After 24h incubation to allow the attachment of cells, the medium was removed and the cells were washed once with 1 mL of the pre-treatment media: complete MEM; complete DMEM; or HS-5 conditioned DMEM. Then the cells were incubated at 37˚C in 1 mL of the pre-treatment media for desired time. After the incubation, the cells were washed once with 1 mL FBS-free medium. The cells were applied with 1 mL FBS-free medium containing Nffy(p) at 0.4 mg/mL and incubated at 37˚C for 4h. The nanonets/hydrogels were collected as mentioned above.

*Gel electrophoresis for protein mass spectrometry*: 18 µL of each sample were mixed with 12 µL of 2X Laemmli loading buffer. The solution were mixed and incubated at 95 ˚C for 5 min. 15 µL of the solution were used for SDS-PAGE. Precast 4–20% gel in Tris-HCl (10 well, 30 µl) were used. The gel was run at constant voltage of 200 V. For protein mass analysis, the gel was stained by Coomassie and each lane was cut into three sections with molecular weight ranges at: 250-80; 80-40; 40-10 kDa. The samples were placed in eppendorf tube and sent to Taplin Mass Spectrometry Facility for analysis.

*Mass spectrometry analysis and data base search:* The procedure details used by the Taplin Mass spectrometry Facility is described as followings: Excised gel bands were cut into approximately 1 mm^3^ pieces and subjected to a modified in-gel trypsin digestion procedure. Gel pieces were washed and dehydrated with acetonitrile for 10 min. followed by removal of acetonitrile. Pieces were then completely dried in a speed-vac. Rehydration of the gel pieces was done with 50 mM ammonium bicarbonate solution containing 12.5 ng/μl modified sequencing-grade trypsin at 4 °C. After 45 min., the excess trypsin solution was removed and replaced with 50 mM ammonium bicarbonate solution to just cover the gel pieces. Samples were then placed in a 37 °C room overnight. Peptides were later extracted by removing the ammonium bicarbonate solution, followed by one wash with a solution containing 50% acetonitrile and 1% formic acid. The extracts were then dried in a speed-vac (~1 hr). The samples were then stored at 4 °C until analysis. On the day of analysis the samples were reconstituted in 5–10 μl of HPLC solvent A (2.5% acetonitrile, 0.1% formic acid). A nano-scale reverse-phase HPLC capillary column was created by packing 5 μm C18 spherical silica beads into a fused silica capillary (125 μm inner diameter × ~20 cm length) with a flame-drawn tip. After equilibrating the column each sample was loaded via a Famos auto sampler onto the column. A gradient was formed and peptides were eluted with increasing concentrations of solvent B (97.5% acetonitrile, 0.1% formic acid). As peptides eluted they were subjected to electrospray ionization and then entered into an LTQ Velos ion-trap mass spectrometer (ThermoFisher). Peptides were detected, isolated, and fragmented to produce a tandem mass spectrum of specific fragment ions for each peptide. Peptide sequences (and hence protein identity) were determined by matching IPI and UNIPROT protein databases with the acquired fragmentation pattern by the software program, SEQUEST (ThermoFisher). Spectral matches were manually examined and carry over proteins and common contaminants (such as keratins and trypsin) were removed. The resultant lists of proteins and peptides identified by SEQUEST and provided by the facility.

*Additional control experiment procedure (“2h N w/o HeLa”):*

For collection of nanonets, the dephosphorylation enzyme (ALPP) was applied with 1mL of complete medium containing precursor at 560µM (diluted from a 20X stock solution of the precursor in PBS buffer). After 2h incubation at 37˚C, the dish were taken out of incubator and placed in 4˚C cool room for 5 min. The dish was tilted and knocked on bench to collect the formed nanonets/hydrogels in medium. The nanonets/hydrogels were immediately frozen at -80˚C.

*Results for “2h N w/o HeLa”:*

While “2h N” trials have 161 and 181 identified proteins, and “2h CM” trials have 128 and 108 proteins, we found only 144 identified proteins in the “2h N w/o HeLa” trial, which is significantly less than the previous trials with HeLa cell incubated in. The lack of identified proteins is expected because the absence of HeLa cells in this control experiment can result in no cancer secretome. This additional control experiment proves that even though there may be concentrating effect of nanonets, the nanonets formed on the cancer cell surface still trap enough secretome to offset the perturbation of the medium.

We also analyzed the overlap of identified proteins between “2h N w/o HeLa”, “2h N” and “2h CM”. The average overlap between “2h N w/o HeLa” and “2h N” is 84 proteins, 30%. The average overlap between “2h N w/o HeLa” and “2h CM” is 63 proteins, 37%.

**S1 Video.** Demonstration of the collection of nanonets.

**S2 Video.** Demonstration of the collection of medium.
